# A Comparative Study of Cavitation Characteristics of Nano-Fluid and Deionized Water in Micro-Channels

**DOI:** 10.3390/mi11030310

**Published:** 2020-03-16

**Authors:** Tao Li, Bin Liu, Jinzhi Zhou, Wenxuan Xi, Xiulan Huai, Hang Zhang

**Affiliations:** 1 Institute of Engineering Thermophysics, Chinese Academy of Sciences, Beijing 100190, China; litao@iet.cn (T.L.); caszhou@163.com (J.Z.); xiwenxuan@iet.cn (W.X.); 2 University of Chinese Academy of Sciences, Beijing 100190, China

**Keywords:** cavitation, nano-fluid, micro-channel, numerical analysis

## Abstract

Hydrodynamic cavitation has been widely applied in micro-fluidic systems. Cavitating flow characteristics are closely related to the fluid properties. In this paper, the cavitation characteristics of Cu nano-fluid in micro-channels were numerically investigated and compared with those of the deionized (DI) water. The mathematical model was verified by comparing the numerical results with the experiment observation. The curved orifice (*R* = 0.3 mm) was found to have the highest efficiencies of cavitation for both fluids. With the increase of inlet pressure, cavitating jet lengths of the two fluids significantly increased. While, the cavitating jet length of the nano-fluid was shorter than that of the DI water at the same inlet pressure. The cavitation inception number of the DI water and nano-fluid were approximately 0.061 and 0.039, respectively. The results indicate that the nano-particles played negative effects on the cavitation inception. In addition, with the decrease of outlet pressure, the cavitation strength gradually increased and the mass flow rate remained nearly unchanged at the same time.

## 1. Introduction

Cavitation is a complex two-phase flow that is caused by a sudden decrease of pressure in the liquid. The growth and collapse of cavities generate an extremely high level of temperature and pressure impulse, which induces a series of complicated physical and chemical effects [1,2]. In recent years, it has been found that cavitation in the micro-fluidic system can be widely applied in many fields, such as heat transfer enhancement, chemical engineering, water treatment, and nano-materials dispersion [3,4,5,6,7,8]. The characteristic of liquid-gas phase transition in the micro-fluidic channel can be significantly different from which in a macro-scale channel because of the dimension limitation. From previous experimental and numerical studies, the characteristics of cavitating flow in the micro-fluidic system have been widely investigated [9]. Mishra et al. [10] experimentally investigated hydrodynamic cavitation in a silicon micro-channel. The results showed that the cavitation inception number was much smaller than that obtained from previous studies on larger orifices. However, choking cavitation was observed to be independent of any pressure or velocity scale effects. Ghorbani et al. [11] studied the cavitating flows between the micro- and macro-scale channels, and results showed that the pressure profile and vapor phase distribution exhibited different features. The static pressure dropped to negative values (tensile stress) in micro-channels, while the minimum static pressure in mini-channels was found to be equal to vapor saturation pressure. Additionally, the higher velocity magnitudes, especially at the outlet, were visible in the micro-channels. It can be seen that, in spite of the geometric similarity, the inception of cavitating flow did not meet the demand of the Newton criterion of dynamic similarity. It was believed that the amount of nuclei in the low pressure zone was the critical factor, which results in the difference of cavitating flow between the micro- and macro-scale. The silicon based high pressure cavitation micro-fluidic system was investigated [12]. The results showed that cavitation could be decreased and eliminated at a sufficiently high backpressure, and it was restricted to the vena contracta in orifice micro-channels. Pennathur and Peles [13] studied cavitation in micro-scale devices that cascaded of micro-pump blades. With increasing mass flow, the size of the cavitation zone grew about 50% being more slowly than predicted by theory. Mishra and Peles investigated the cavitating flow of deionized water (DI water) through various micro-orifices and micro-channels [14]. Multifarious cavitating flow patterns, including incipient, choking, and super-cavitating, were detected. The results displayed that, in spite of several parameters, the trends were similar at both scales, and the flow patterns were different for micro- and conventional scale orifices. For the super-cavitating flow patterns, vapor cavity was encompassed by liquid in the micro-channel; however, it was found inside the vapor pocket in the conventional scale orifices. 

The computational fluid dynamics (CFD) method plays an important role in the investigation of cavitation. Thus far, the full cavitation model [15], Schner–Sauer model [16], and Zwart-Gerber-Belamri model [17] are commonly used to simulate the hydraulic cavitating flow. The full cavitation model has been employed to simulate the cavitating flows in pumps and inducers. The effects of turbulent fluctuations and non-condensable gases were also considered in the model. The numerical simulation results precisely predicted the cavitating flow trends and the size, location, and shape of the cavitation zone [18]. The cavitation flows over a two dimensional (2-D) hydrofoil and an axisymmetric ogive were simulated, and the nuclei density solutions that were obtained by the simulation showed good consistency with measurements [19]. By using Zwart-Gerber-Belamri model, the cavitating flow through a micro-orifice was investigated. The results showed that the vapor cavity region increased with the rise of pressure and the entire micro-orifice wall could be covered by vapor. Additionally, it was recommended that the maximum l/d of a micro-orifice is about 1 [20]. 

In recent years, nano-fluid has been more widely applied in micro-fluidic systems, which is a multiphase fluid that contains nanometer-sized solid particles [21,22,23]. Because of the existence of nano-paritcles, the physical properties of nano-fluid, such as the viscosity, density, surface tension, and thermal conductivity, are significantly different when compared with the base liquid [24,25,26]. Thus, cavitation would be affected by the addition of nano-particles. Gu et al. [27] applied the acoustic method to experimentally study the effects of SiO_2_ nano-particles on cavitation inception. The temperature and particle size were variables and the dimensionless free energy of the critical bubble was calculated in the experiments. The results showed that the SiO_2_ particles always promoted the cavitation inception. However, the increase of particle concentration further promoted the cavitation, while the particle size had little effect. Bidhendi et al. [28,29] examined the effects of SiO_2_ nano-particles on initiation of cavitation in a centrifugal water pump. In the research, the nano-particle concentration, size, and fluid temperature were changed. It was found that SiO_2_ nano-particles could decrease the rate of cavitation growth. However, until recently, there is relatively little study of nano-fluid cavitation in the micro-fluidic system. In this paper, the computational fluid dynamics (CFD) method was employed for simulating the cavitating flows of nano-fluid and deionized (DI) water in micro-channels. The results were validated by comparison with experimental observations. The differences of cavitation dynamics between the nano-fluid and DI water were discussed in detail. Additionally, the effects of the orifice shape, the inlet pressure, and outlet pressure on cavitation were analyzed. This work is aimed to provide useful insights for the application of nano-fluid cavitation in MEMS and other devices with micro-channels.

## 2. Numerical Models 

### 2.1. Governing Equations

The Reynolds Averaged Navier-Stokes (RANS) approach was used to analyze the cavitation flow field in the micro-channel. The mass conservation equation is expressed as:

(1) $$\frac{\partial }{\partial x_{i}}(\rho u_{i})=0$$

Here, *ρ* and *u_i_* are the fluid density and average velocity component, respectively. The Reynolds-averaged momentum conservation equation can be expressed as:

(2) $$\frac{\partial }{\partial x_{j}}(\rho u_{i}u_{j})=-\frac{\partial p}{\partial x_{i}}+\frac{\partial }{\partial x_{j}}[\mu \frac{\partial u_{i}}{\partial x_{j}}+R_{ij}]$$

Here, *μ* stands for the kinematic viscosity and *R_ij_* is the Reynolds stress tensor. In this study, the volume of fluid (VOF) model tracks the volume fraction of the each fluid throughout the domain. The tracking of interface between phases is accomplished by the solution of a continuity equation for the volume fraction of one (or more) of the liquid phases, and this equation has the following form:

(3) $$\nabla \cdot (\alpha_{q}U_{q})=\frac{1}{\rho_{q}}(m_{pq}-m_{qp})$$

Here, *m_qp_* is the mass transfer rate of the liquid phase *q* to the vapor phase *p*, and *m_pq_* is the mass transfer rate of the vapor phase *p* to the liquid phase *q*. *U_q_* is the mean velocity of the *q* phase. The liquid phase volume fraction is computed, based on the following equation:

(4) $$\alpha_{q}+\alpha_{p}=1$$

### 2.2. Turbulence Modeling

So far, little study has been conducted on the effects of turbulence models on cavitating flow in the micro-channels. In this study, the Standard *k*-*ω* model, *k*-*ω* Shear-Stress Transport (SST) model, Standard *k*-*ε* model, and Realizable *k*-*ε* model are selected. The transport equations of Standard *k*-*ω* model are shown, as follows

(5) $$\frac{\partial }{\partial x_{i}}(\rho ku_{i})=\frac{\partial }{\partial x_{j}}(\Gamma_{k}\frac{\partial k}{\partial x_{j}})+G_{k}-Y_{k}+S_{k}$$

Additionally

(6) $$\frac{\partial }{\partial x_{i}}(\rho \omega u_{i})=\frac{\partial }{\partial x_{j}}(\Gamma_{\omega }\frac{\partial \omega }{\partial x_{j}})+G_{w}-Y_{\omega }+S_{\omega }$$

The turbulence kinetic energy *k* and the specific dissipation rate *ω* are obtained from these two transport equations. In these equations, *G_k_* represents the generation of turbulence kinetic energy due to the mean velocity gradients. *G_ω_* represents the generation of specified dissipation rate. *Y_k_* and *Y_ω_* represent the dissipation of *k* and *ω* due to turbulence. S*_k_* and S*_ω_* are user-defined source terms. Γ*_k_* and Γ*_ω_* represent the effective diffusivity of *k* and *ω*, respectively, which are expressed as:

(7) $$\Gamma_{k}=\mu +\frac{\mu_{t}}{\sigma_{k}}$$

(8) $$\Gamma_{\omega }=\mu +\frac{\mu_{t}}{\sigma_{\omega }}$$

The *k*-*ω* Shear-Stress Transport (SST) model is based on the standard *k*-*ω* model and the turbulent viscosity *μ_t_* is amended.

The transport equations of standard *k*-*ε* model are shown, as follows:

(9) $$\frac{\partial }{\partial x_{i}}(\rho_{m}ku_{i})=\frac{\partial }{\partial x_{j}}\left[(\mu_{m}+\frac{\mu_{t}}{\sigma_{k}})\frac{\partial k}{\partial x_{j}}\right]+G_{k}+G_{b}-\rho_{m}\varepsilon -Y_{M}+S_{k}$$

(10) $$\frac{\partial }{\partial x_{i}}(\rho_{m}\varepsilon u_{i})=\frac{\partial }{\partial x_{j}}\left[(\mu_{m}+\frac{\mu_{t}}{\sigma_{\varepsilon }})\frac{\partial \varepsilon }{\partial x_{j}}\right]+C_{1}{}_{\varepsilon }\frac{\varepsilon }{k}(G_{k}+C_{1}{}_{\varepsilon }G_{b})-C_{2\varepsilon }\rho \frac{\varepsilon^{2}}{k}+S_{\varepsilon }$$

Here, *G_b_* is the generation of turbulence kinetic energy due to buoyancy and *Y_M_* represents the contribution of the fluctuating dilatation in compressible turbulence to the overall dissipation rate. The model of Realizable *k*-*ε* model is based on the standard *k*-*ε* model and the mathematical constraint is used to improve the performance of the model. 

### 2.3. Cavitation Model

In the study, the Schner-Sauer model was employed. The mass transfer source terms connected to the growth and collapse of the vapor bubbles, which are based on the Rayleigh-Plesset equations and defined as:

(11) $$m_{qp}=\frac{\rho_{p}\rho_{q}}{\rho }\alpha_{p}(1-\alpha_{p})\frac{3}{R_{B}}\sqrt{\frac{2}{3}\frac{\left(P_{p}-P\right)}{\rho_{q}}},\;\mathrm{when}\;P\leq \;P_{v}$$

(12) $$m_{qp}=\frac{\rho_{q}\rho_{p}}{\rho }\alpha_{p}(1-\alpha_{p})\frac{3}{R_{B}}\sqrt{\frac{2}{3}\frac{\left(P-P_{p}\right)}{\rho_{q}}},\;\mathrm{when}\;P_{v}\leq \;P$$

Here, *R_B_* means the bubble radius. 

## 3. Results and Discussion

### 3.1. Model Validation

**Figure 1:**
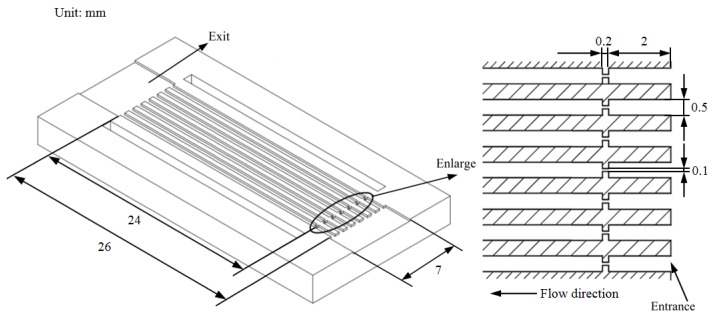
The structure of the micro-channels.

In this section, the numerical results were first compared with experimental data [30] to prove the accuracy and reliability of the model. In the previous experiment, a copper-based plate with rectangular micro-channels was fabricated, which consisted of seven parallel channels. Each of the channels was 26 mm long, 0.5 mm wide, and 0.2 mm deep. At 2 mm downstream of the entrance, the rectangle orifice with the width of 0.1mm and length of 0.2mm was designed, as shown in Figure 1. The DI water flowed through channels that were driven by the pump and cavitation was induced by a sudden drop of liquid pressure at the orifice.

**Figure 2:**
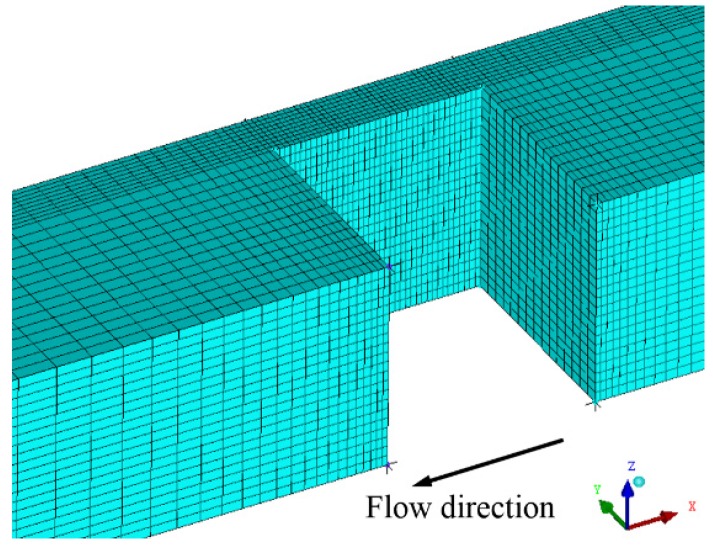
The micro-channel geometry in the simulation.

In the simulation, one channel was selected, so that the amount of calculation can be effectively reduced, and only a half of the channel geometry was modeled due to the presence of symmetry planes. We employed structured quadrilateral meshes to capture the vapor phase, as the micro-channel was not geometrically complex, as shown in Figure 2. The mesh was generated with ICEM CFD software. In order to evaluate the grid dependency of the studied geometry, three different cell numbers were compared: 158,730, 335,223, and 484,290, respectively. 

**Figure 3:**
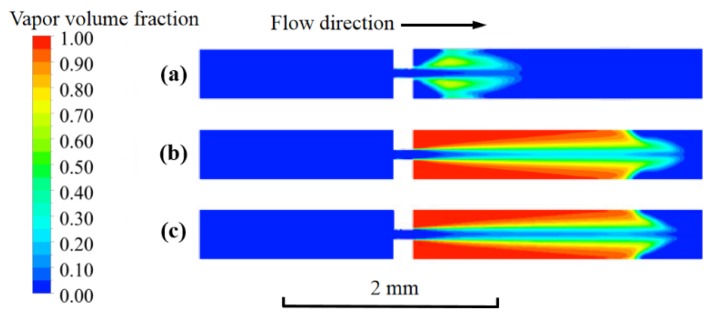
The vapor fraction at the cross-section *z* = 0.1 mm of the micro-channel, (**a**) Grid 158,730 cells; (**b**) Grid 335,223 cells; and, (**c**) Grid 484,290 cells.

The boundary conditions of the inlet and outlet pressure were set to be 355 and 8.5 kPa in accordance with the experimental parameters [30]. The cavitation flow field was obtained while using the commercial CFD software ANSYS CFX release 14.0. The finite volume method was utilized to perform a discrete solution for the governing equations. The second order upwind scheme was used to discretize the mass, momentum, turbulent quantities and vapor transport equations. The pressure corrections were computed using the body force weighted Pressure Staggering Option (PRESTO!) scheme. For pressure-velocity coupling, the semi-implicit method for pressure linked equations (SIMPLE) algorithm was employed. The micro-channel walls were treated as non-slip boundaries with standard wall functions. In the simulation, convergence was assumed when the residuals dropped below a value of 10^−4^. Figure 3 depicts the calculated vapor fractions at the cross-section *z* = 0.1 mm of the micro-channel. It can be observed that the difference between the vapor fractions became negligible as the number of cells more than 335,223. Thus, the grid was applied for all cases in this paper.

When fluid flowed through the micro-orifice, some specific features, including cavitation clouds and high speed vapor-liquid jet, would occur. Therefore, a critical task of suitable turbulence model was to capture the dynamics of cavitation growth and collapse correctly in a very small scale. Table 1 provides the calculated mass flow rate with different turbulence models. As observed, the solution of the standard *k*-*ɷ* model was quite close to the experimental data, with a maximum of 1.5% error. By comparison, for the Standard *k*-*ε* and Realizable *k*-*ε* model, the errors of mass flow rate increased to about 7.1% and 7.3%, respectively.

**Figure 4:**
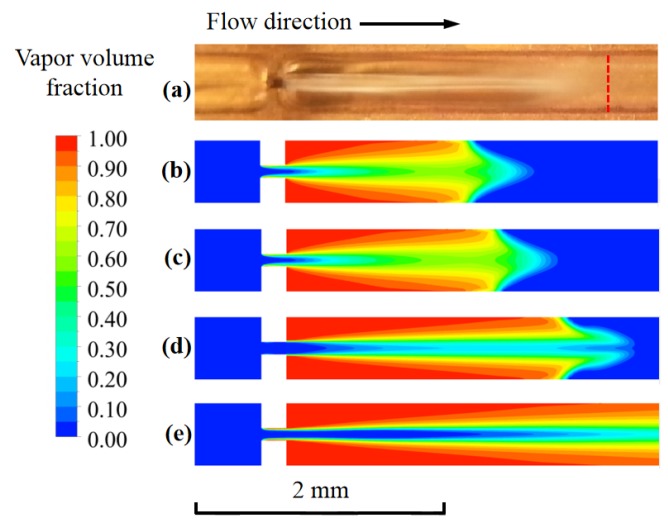
The comparison of vapor-liquid distribution between the experimental data [30] and simulation results, (**a**) experimental result; (**b**) standard *k*-*ε* model; (**c**) realizable *k*-*ε* model; (**d**) standard *k*-*ω* model; and (**e**) k-*ω* Shear-Stress Transport (SST) model.

The simulated vapor-liquid distributions at the cross-section *z* = 0.1 mm by various turbulence models were compared with the experimental result, as displayed in Figure 4. The calculated vapor-liquid distribution by Standard *k*-*ω* model showed better agreement with the experimental result. For cavitation that occurred in a confined space, a shear flow boundary layer that formed by a large velocity gradient existed between the vapor and liquid phase. Meanwhile, the Reynolds number was relatively low downstream of the orifice. The Standard *k*-*ω* model considers the low Reynolds number effects and shear flow diffusion. Thus, it is more suitable for the wall-bound and jet flows calculation as the cavitating flow in the micro-channel.

**Figure 5:**
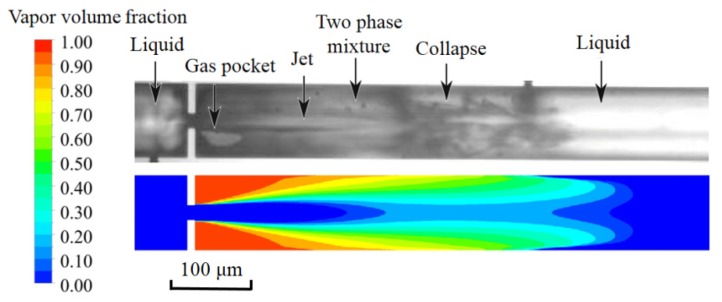
The comparison of vapor-liquid distribution with the experimental observation [31].

Additionally, the calculation result was compared with the experimental result that was obtained by Rooze et al. [31], as shown in Figure 5. It could be seen that a cavitating jet formed when the fluid flowed through the micro-orifice. The liquid jet was surrounded by twin vapor bubbles near the orifice. The liquid–gas two phase mixtures were observed at downstream of the liquid jet. With the recovery of pressure, those cavitation bubbles collapsed and turned into liquid. The comparison between the present results and the experimental data from literature further verified the validity of the proposed numerical method. 

### 3.2. Cavitating Flow Characteristics of Nano-fluid and DI Wwater

**Figure 6:**
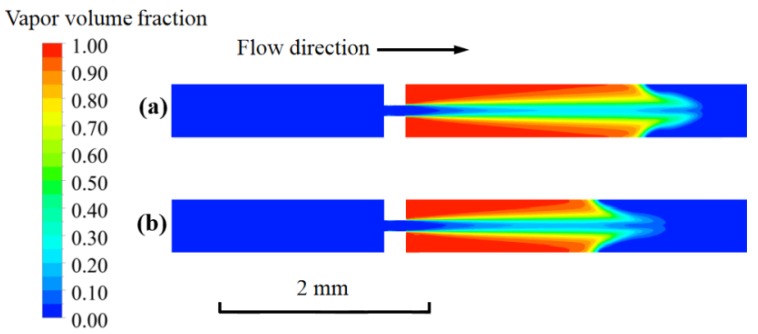
The vapor-liquid distribution of the fluids: (**a**) deionized (DI) water; and, (**b**) nano-fluid.

In the work, the Cu nano-fluid with 3% volume fraction and particle size of 100 nm was selected. The density and viscosity of the nano-fluid were 1250 kg/m^3^ and 1.18 × 10^−3^ kg/m·s, respectively. The inlet and outlet pressure were set to be 355 and 8.5 kPa, respectively. Figure 6 shows the cavitating flow fields of the nano-fluid and DI water near the rectangle orifice. It can be observed that the cavitation jets for both nano-fluid and DI water were quite similar. However, the cavitating jet length of nano-fluid (2 mm) was a little shorter than the length of the DI water, which is in agreement with the experiment that was conducted by Medrano [32]. 

**Figure 7:**
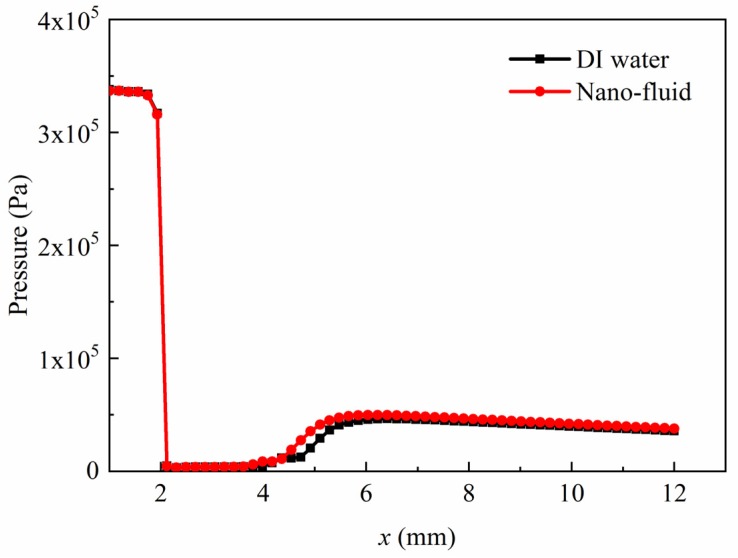
The pressure distributions along centerline on the symmetry plane.

Figure 7 displays the pressure profiles of nano-fluid and DI water along the centerline on the symmetry plane. When the fluids flowed through the orifice, a sudden pressure drop below the saturated vapor pressure (3540 Pa) was observed, which caused the vapor-liquid phase transition. Downstream of the orifice, the pressure gradually recovered to about 8.5 kPa. The pressure recovery of the nano-fluid was a little bit faster when compared with that of the DI water due to a higher surface tension. Thus, the length of nano-fluid cavitating jet was shorter due to an earlier vapor bubble collapse.

**Figure 8:**
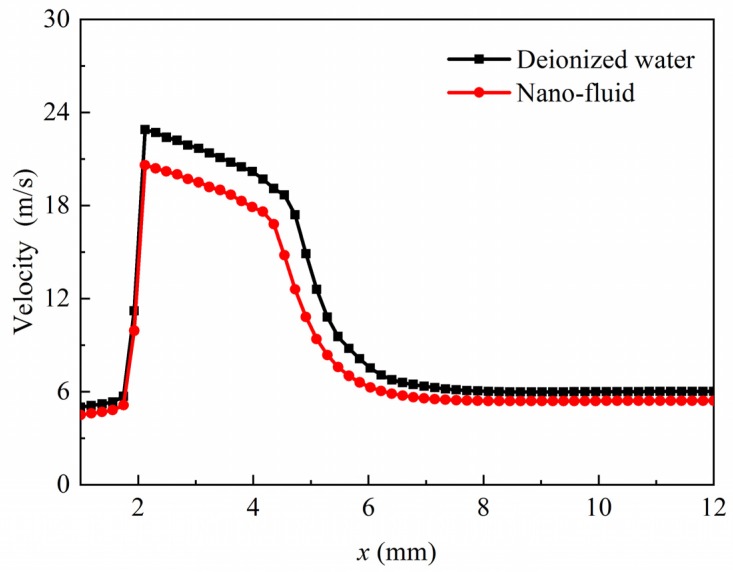
The velocity distributions at centerline on the symmetry plane.

Figure 8 shows the velocities along the centerline on the symmetry plane. There was a significant increase in flow velocity at the orifice, due to the sudden decrease of the flow area and the cavitation phase change. The maximum cavitating jet velocity of the DI water reached approximately 22.9 m/s, which was a little higher than the jet velocity of the nano-fluid (20.4 m/s). It indicated that the cavitation intensity of nano-fluid could be about 12% lower. After flowing through the orifice, the velocities of both fluids began to decline. At the very beginning, the velocity decreased relatively slower in the cavitation zone, because of the presence of the vapor phase. By comparison, a more significant decline of the velocity occurred after the cavitation collapse.

### 3.3. Effects of the Orifice Structure

**Figure 9:**
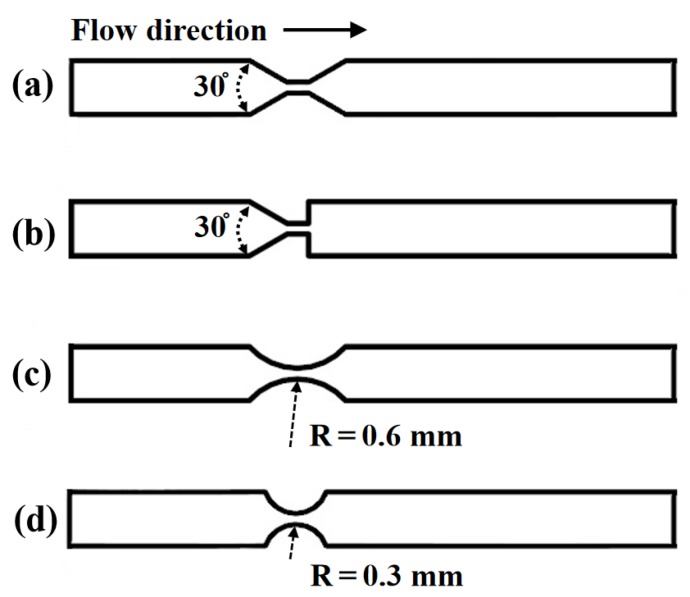
The orifice structures, (**a**) converging-diverging orifice, (**b**) converging orifice, (**c**) curved orifice (*R* = 0.6 mm), and (**d**) curved orifice (*R* = 0.3 mm).

In this section, the effects of the orifice structures on cavitation characteristics were analyzed. Besides the rectangle orifice, four orifices as the converging-diverging orifice, the converging orifice, and the curved orifices (*R* = 0.3 and 0.6 mm) were selected, in which are shown Figure 9. The micro-channel width to the orifice minimum width ratio was *W*_c_/*W*_omin_ = 5. For the converging-diverging orifice, the contraction and divergence angles are both 30 degrees, as shown in Figure 9a. Additionally, the contraction angle of the converging orifice is 30 degrees (see Figure 9b). The micro-channels with curved orifices are shown in Figure 9c,d, and the radii are 0.6 and 0.3 mm, respectively.

The inlet and outlet pressure in the calculation were 355 kPa and 8.5 kPa, respectively. Table 2 shows the mass flow rate of the DI water and nano-fluid under different orifices. Generally, the mass flow rate of the nano-fluid was approximately 12% lower than that of the DI water for all of the different orifices. Meanwhile, for both fluids, the minimum flow rates were under the rectangle orifice attributing to a more significant local resistance loss. When compared with the rectangle orifice, the highest mass flow rates were under the curved orifices, being about 30% higher. However, with the increase of the radius, the mass flow rate was almost kept unchanged for the curved orifice. 

**Figure 10:**
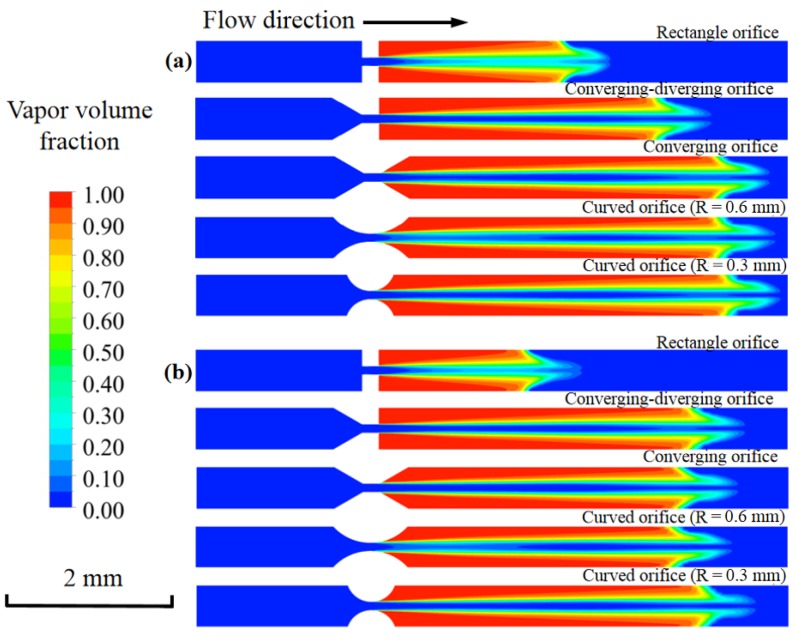
The vapor volume fraction with various orifice structures: (**a**) DI water and (**b**) nano-fluid.

Figure 10 shows the vapor volume fraction of the DI water and nano-fluid under various orifice structures. For both fluids, the longest cavitating jet lengths were observed under the curved orifice with *R* = 0.3 mm. On one hand, the flow resistance under the curved orifice (*R* = 0.3 mm) was much lower. On the other hand, the smooth curve of the orifice led to slow pressure recovery downstream of the channel. Thus, the mass flow rate and the cavitation vapor fraction were obviously higher. Additionally, it was found that the cavitating jet length of DI water was a little bit longer than the length of nano-fluid at each orifice structure. The addition of nano-particles increased the viscosity and surface tension of the base fluid, which is believed to have negative effects on cavitation inception.

### 3.4. Effects of the Inlet Pressure

**Figure 11:**
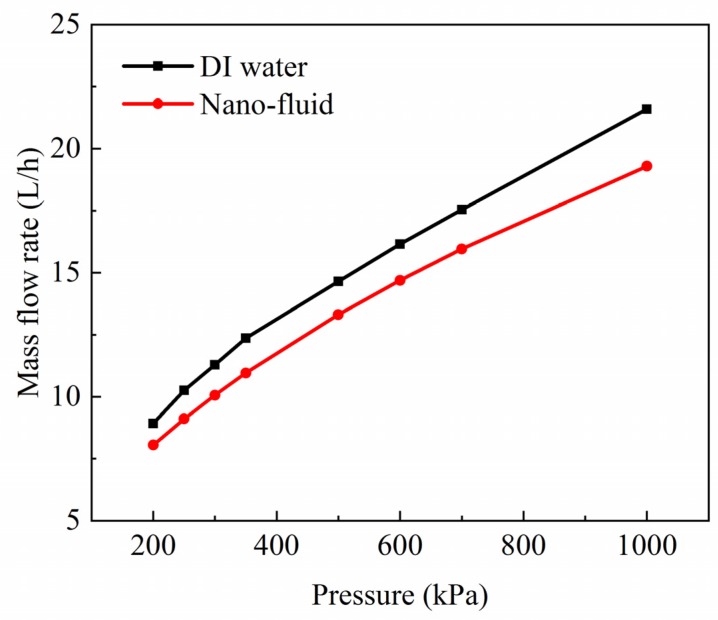
Mass flow rates of the nano-fluid and DI water under different inlet pressures.

In this section, the effects of the inlet pressure on the DI and nano-fluid cavitation were analyzed. The structure of curved orifice (*R* = 0.3 mm) was selected because of the highest cavitation efficiency. The outlet pressure was set to be 8.5 kPa. Figure 11 displays the mass flow rates of the DI water and the nano-fluid with different inlet pressures. For both fluids, the mass flow rates grew almost linearly with the increase of inlet pressure. While, for the same inlet pressure, the mass flow rate of the nano-fluid was always found to be lower than that of the DI water. Furthermore, the mass flow rate difference between the two fluids got higher with the growth of inlet pressure. 

The cavitation inception number is an important dimensionless parameter, which is used to characterize the initial and critical state of cavitation. The cavitation inception number is defined, as follows:

(13) $$\sigma =\frac{P_{out}-P_{v}}{\frac{1}{2}\rho U_{o}^{2}}$$

**Figure 12:**
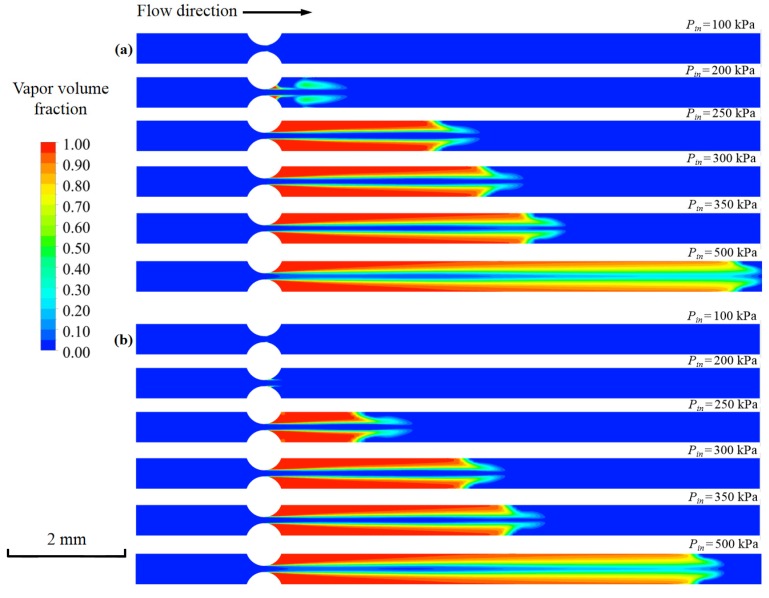
The vapor volume fraction with various inlet pressures: (**a**) DI water and (**b**) nano-fluid.

Here, *P_out_* is the outlet pressure, *P_v_* is the vapor pressure, and *U_o_* is the mean velocity at the orifice. Figure 12 shows the vapor volume fractions of two fluids with different inlet pressure. The cavitation inception of nano-fluid could be observed at *σ* = 0.039 (inlet pressure of 200 kPa). In contrast, *σ* for the DI water was approximately 0.064. Thus, the nano-fluid was more difficult to generate cavitation than the pure water. Additionally, the length of the nano-fluid was found to be shorter when compared with the DI water for the same inlet pressure. With the increase of inlet pressure, the length of cavitating jet increased significantly. When the inlet pressure grew to 500 kPa, cavitating jet lengths of both fluids increased to about 8 mm up to 30% of the total channel length. Thus, increasing the inlet pressure is one of the effective ways to promote the cavitation intensity. 

### 3.5. Effects of the Outlet Pressure

The outlet pressure of 5, 8.5 and 12 kPa were selected in order to study their effects on cavitation inside the micro-channel with the curved orifice (*R* = 0.3 mm). The inlet pressure was 350 kPa in the calculation. Table 3 shows the mass flow rates of the DI water and nano-fluid under different outlet pressures. For both of the fluids, the mass flow rate was found to be independent of the outlet pressure. When the outlet pressure increased from 5 to 12 kPa, the mass flow rates of the both fluids remained nearly unchanged.

**Figure 13:**
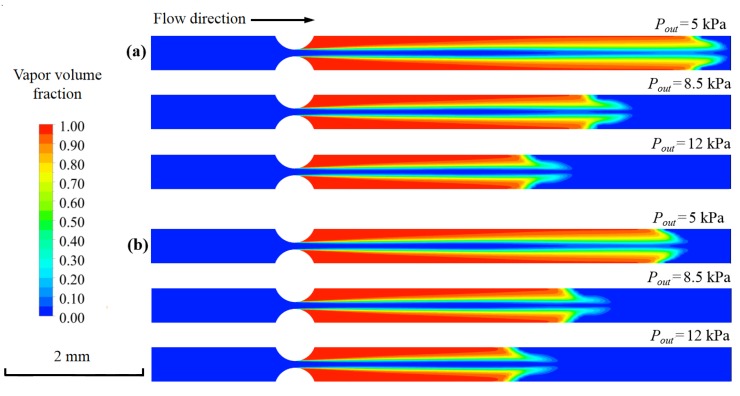
The vapor volume fraction with various outlet pressure: (**a**) DI water and (**b**) nano-fluid.

Figure 13 shows the vapor volume fraction of the DI water and nano-fluid under different outlet pressures. As the outlet pressure decreased from 12 kPa to 5 kPa, the cavitating jet length gradually increased. Meanwhile, for the same outlet pressure, the vapor volume fraction of the DI water was a little higher than that of the nano-fluid. The result indicates that, by decreasing the outlet pressure, the higher cavitation intensity can be acquired without the change of mass flow rate.

## 4. Conclusions

In this paper, the cavitation characteristics of the DI water and nano-fluid inside micro-channels with orifices were numerically investigated. The calculated results were compared with the experimental data, which proved the stability and reliability of the present model. The standard *k*-*ω* model is more suitable for micro-channel cavitation flow simulation. As to the rectangle orifice, the cavitating jet length and the maximum jet velocity of the nano-fluid were approximately 12% lower than those of the DI water when the *P_in_* and *P_out_* were 350 and 8.5 kPa, respectively. It proves that nano-particles played a negative effect on the cavitation inception. Subsequently, the effects of the orifice structure, inlet, and outlet pressure were discussed in detail. For both fluids, the curved orifice (*R* = 0.3 mm) had the highest cavitation efficiency and mass flow rate. With the increase of inlet pressure, the cavitating jet length and mass flow rate of two fluids increased significantly. Meanwhile, the mass flow rate difference between the two fluids became higher as the inlet pressure grows. In addition, the cavitaion inception number of the DI water and nano-fluid were about 0.061 and 0.039, respectively. With the decrease of outlet pressure, the cavitation intensity became stronger without the change of mass flow rate. The present research only considers the case of Cu nano-fluid with 3% volume fraction and a particle size of 100 nm. In the future, the effects of nano-particle type, size, and nano-fluid concentration on cavitation in the micro-channels would be further studied.

## Figures and Tables

**Table 1:** Mass flow rate calculated by different turbulence models.

Items	Mass Flow Rate (L/h)	Error (%)
Experiment	9.6	―
Standard *k*-*ε* model	8.92	7.1%
Realizable *k*-*ε* model	8.90	7.3%
*k*-*ω* SST model	9.07	5.5%
Standard *k*-*ω* model	9.45	1.5%

**Table 2:** Mass flow rate under different structures.

Structure of the Orifice	DI Water (L/h)	Nano-Fluid (L/h)
rectangle orifice	9.45	8.39
converging-diverging orifice	11.56	10.37
converging orifice	11.53	10.29
curved orifice (*R* = 0.6 mm)	12.23	10.90
curved orifice (*R* = 0.3 mm)	12.36	10.95

**Table 3:** Mass flow rate under different outlet pressures.

Outlet Pressure (kPa)	DI Water (L/h)	Nano-Fluid (L/h)
5	12.36	10.95
8.5	12.32	10.93
12	12.34	10.94
